# Impact of quality management systems in the performance of educational centers: educational policies and management processes

**DOI:** 10.1016/j.heliyon.2020.e03824

**Published:** 2020-04-27

**Authors:** F. Díez, A. Villa, A.L. López, I. Iraurgi

**Affiliations:** a Department of Innovation and Educational Organization, University of Deusto, Spain; b University of Deusto, Spain; c University of the Basque Country, Spain; d Department of Personality, Evaluation and Psychological Treatment, University of Deusto, Spain

**Keywords:** Education, Assessment methods, Educational quality, EFQM Excellence model, Quality management systems, Quality models

## Abstract

In this paper, the findings of an I + D + i research are presented. In this study, an analysis was conducted to assess 14 educational centers where in one of two distinct quality systems had been implemented: the EFQM (European Foundation Quality Management) and el Proyecto de Calidad Integrado (PCI)—the Integrated Quality Project—promoted by the Horrêum Foundation (Álvarez and Santos, 2003; Villa and Marauri, 2004).

The EFQM was first used by businesses before being recently transferred to the academics. It comprised nine factors that were translated in an educational context: leadership, policy and strategy, people, alliances and resources, processes, impact on people, impact on clients, impact on society, and key impacts of an organization. The first five factors examine the way activities are carried out and improved, and the final four focus on the impact, i.e., the effect of the organization's activities. Improvement is achieved through learning and innovation.

The PCI (Muñoz and Sarasúa, 2005) has its educational origins in the Effective School Improvement model. Seven factors are analyzed (Sarasola et al., 2003; Villa et al., 2004): institutional approach, organizational structures, relationships and living together, counseling and tutoring, curriculum, family and the community (Martínez and Galíndez, 2003), and management and services.

The study looks at the impact that the two aforementioned quality systems (EFQM and PCI) have had on educational centers. The term “impact” is understood as the changes experienced both inside and outside an educational center. It must be sustainable overtime, considering the changes and effects achieved, as evidence of improvement.

The quantitative analysis focuses on two dimensions. The first addresses three key factors of educational policy: educational planning, communication, and support and rewards for teachers. The second comprises three factors linked to management processes in educational institutions: organizational climate, teaching and learning processes, and relationships with the community.

## 1 Introduction

The evaluation of educational impact aims to assess whether a program has produced desired effects on people, homes, and the institutions that are involved (Cook and Lineberry, 2016). In other words, the purpose is to quantify the benefits and evaluate whether they are a direct cause of the program that has been developed (Aedo, 2005). Thus, the evaluation of impact must identify if there is a cause–effect relationship between the implemented program and the results expected and achieved. This is crucial to distinguish between the results that are caused by the program and the ones that are linked to some other external factors that could also affect the results during the program's implementation (Baker, 2000).

With this purpose in mind, this study addresses several factors of the two quality management systems: the Proyecto de Calidad Integrado (PCI) (Integrated Quality Project) (Álvarez and Santos, 2003, Muñoz and Sarasúa, 2005, Villa and Marauri, 2004) and the European Foundation Quality Management Model (EFQM). Both systems are analyzed using the following double dimension structure (Díez, 2015):

### 1.1 Educational planning

Educational planning has been a factor of efficacy and school improvement by different authors (Epstein, 2018; Bernhardt, 2013; Davies and Ellison, 2003). Research in educational system reforms underlines that in the last two decades, short-term planning has increased because school planning consists of target-setting plans with the purpose of improving previously established standards. However, short-term planning needs to be incorporated in a longer-term planning framework, i.e., over a 3–5 year period, that supports the strategic development of a school (Davies and Ellison, 2003).

Considering that the aim is to achieve school improvement, evidence shows that educational planning focuses on contributing to the establishment of organizational structures that sustain educational change, particularly in enhancing student learning outcomes and, at the same time, fostering a school's capacity for managing change (Hoque et al., 2011; Stoll, 2009). Therefore, the first step is to understand the organizational climate and structures to build up management structures that can sustain capacity building and educational change. Analysis concentrates on organizational culture, conditions, and strategies; teaching and learning practices; and a school's capacity for decision-making and problem solving (Hopkins et al., 2014).

Based on this analysis, management strategies are formulated by looking closely at the vision and mission of a school (Vos et al., 2013). Relevance is emphasized in the planning process because the way planning is done is as important as the plan itself. Staff are involved; decisions are agreed upon; and steps for action are arranged so that the plan can be implemented (Ainscow et al., 2000). Thus, planning becomes a collaborative as well as an on-going process for school development.

Planning for changes in quality concentrates on improvement at three organizational levels, linking school priorities with the suitable required to achieve them. At the school level (García and Aguirregabiria, 2006) efforts are concentrated on policies, particularly those related to the mobilization of resources and strategies for professional development to support school improvement. At the working-group level, planning concerns with the details and organization of supporting improvement activities. At the individual level, it addresses the enhancement of school practice (Ainscow et al., 2000; Fullan, 2010). In addition, if the purpose is for school improvement to last for a long time, it needs to challenge not only quality but also equity (Stoll, 2009).

Past research has highlighted the relevance of a principal's role in school improvement (Glatthorn et al., 2016). The strategic planning of principals appears to be significant and to have a positive effect on school improvement (Hoque et al., 2011). The most valued strategic planning roles relate to developing school improvement plans, staying abreast of work, promoting the vision and mission, organizing meetings, and recognizing success.

Besides, principals have a central role in school government. School leaders control the key mechanisms of educational governance systems, promoting autonomy. Such autonomous schools develop decision-making authority in four relevant areas of school improvement; the primary one wherein principal exercise a great deal of autonomy being school budget and the others being curriculum, instruction, and assessment. Moreover, they manage the timing of organizational change by arranging calendars and schedules, and they assume their responsibility for staff professional development, thereby becoming pivotal actors in their own organizational capacity building process (Fullan, 2010; Steinberg, 2014).

### 1.2 Communication

Relationships and communications are key characteristics if quality improvement has to be implemented in schools (Jefferson and Anderson, 2017). Four of the conditions that leaders should guarantee to develop a school atmosphere facilitating transformations are based on interpersonal relations. First, commitment to collaborative planning is required. Second, staff, students, and the community need to be involved in school policies and decisions. Third, effective coordination strategies must be established. Finally, professional development must be a priority (Ainscow et al., 2000). We will explore this last condition in greater detail later in this paper.

According to evidence, the main process of planning, as aforementioned, is participative and involves staff members and other groups in the school life. In addition, information channels are organized so that everyone collaborating in the improvement plan knows what is happening, the way things are being implemented, and the reasons behind the decisions and actions that are taken (Ainscow et al., 2000). As planning is a continuous process, well-informed members are constantly motivated and committed, and their collaboration continues over time and is crucial in helping the school move forward.

Research shows that school leaders guide comprehensive planning, delegate tasks to different teams, and are flexible in improving relationships between teachers and students (Bell, 2018; Stein, 2016). They also accept and understand social needs by enhancing their own quality. Furthermore, school leaders emphasize the responsibilities acquired by working groups and motivate them to do their best to fulfill common goals (Hoque et al., 2011).

### 1.3 Support and rewards for teachers

Teachers who feel recognized have positive feelings about their work, experience high self-esteem and self-confidence, assume authority, and are motivated to work hard (Egeberg et al., 2016). They have a positive attitude and a higher involvement in the organization (OCDE, 2010). As teachers and other professional staff are at the heart of every educational community, support and professional development for teachers is pivotal in any school improvement process.

Several authors (Agudo and Antón, 2004) consider fostering capacity building as critical for school improvement; however, it presents a certain challenge. At an individual level, capacity building is concerned with educators' competencies, resources, and motivation. Although sometimes unknown, individuals and groups have high capacities, which are enhanced by engaging in processes of knowledge and skills development. Teachers and staff who constantly commit to working collectively develop their own capacities while organizational capacities increase. Professional educators involved in capacity building challenge their practices and have the opportunity to do things differently, learn new skills, and improve their teaching to be more effective (Harris, 2011). Moreover, the development of teaching practices appears to have a direct positive effect on students’ learning (Thoonen et al., 2012).

At an organizational level, schools create and maintain the necessary conditions, considering the policies, cultures, and structures that facilitate learning and skill-oriented experiences. They also ensure inter-relationships and synergy for capacity building (Fullan, 2010; Stoll, 2009). Guaranteeing organizational conditions that strengthen school capacity for change is a prerequisite for unifying educators’ professional development with school development (Thoonen et al., 2012).

Moreover, staff reward procedures must link individual and overall needs of a school; otherwise, appraisal measures may lead to the neglect of school development in favor of individual incentives (Mosoge and Pilane, 2014). Besides, professional development plans need to be considered within the school's budget and timetable (Ainscow et al., 2000).

### 1.4 Organizational climate

Educational communities are working to develop an open climate, with policies and structures promoting the involvement of community members, educators, students, families, and members of the wider community (Wang and Degol, 2016). Thus, their commitment toward school success is promoted. Additionally, another particularly relevant factor for school improvement is student participation (Ainscow et al., 2000).

With the aim of developing a collaborative school management, members participate in goal setting, policy-making, budgeting, improvement implementation, and even evaluation (Hopkins et al., 2014). To this end, principals and leaders are supportive so that teachers are genuinely committed to and involved in school development. They build up a conducive environment that fosters teaching and learning in addition to teachers' engagement in self-development (Hoque et al., 2011). Proper time management guarantees a scheduled time to plan and work together with colleagues. Evidence suggests that common planning time, flexible scheduling, and interdisciplinary teams improve students’ achievement, particularly in secondary education (Miller and Rowan, 2006).

Communication and personal interactions are crucial in establishing coordination. Quality interpersonal relationships have strong effects in achieving aims in the context of a school. A positive working atmosphere is valued, wherein coordinators know how to interact with colleagues and develop skills to support working teams to develop changing initiatives. A planned and monitored communication system facilitates dialog about teaching, agreement on common purposes, and interchanging practices and expertise. Thus, cooperative structures positively influence performance and foster a learning-enriched school (Fullan, 2010). Accordingly, efforts made in the following four areas guarantee strategic coordination structures for improvement: the establishment of skillful coordinators, using task groups to get things done, the establishment of communication networks, and facilitating discussion of practice (Ainscow et al., 2000).

### 1.5 Teaching and learning processes

Evidence regarding school improvement demonstrates that educational centers guarantee the conditions that motivate teachers to get involved in learning processes. These challenge their teaching practices to improve students' learning. Staff development programs aim at supporting educators to develop their teaching and analyze students’ learning, as this is the only way to achieve school improvement. Following these clear aims, schools establish a policy for staff development, which is classroom-focused. Besides, they develop structures to become centers for professional learning (Ainscow et al., 2000).

Furthermore, policies for professional development also appear to focus on a school's needs, thereby building a highly trustworthy environment and a framework wherein teachers feel secure and motivated to challenge and exchange their knowledge and practices. The purpose of a policy is to construct an effective learning community with the aim of improving pedagogy and student achievement. Staff development programs address classroom practices, provide opportunities to model instructional approaches, and develop in-depth knowledge on specific class matters. In addition, they are extended and distributed overtime so that sustainable long-term changes can be achieved (Harris, 2011; Leithwood, 2010; Miller and Rowan, 2006). For this to happen, school-level leadership development is crucial (Ingersoll et al., 2018). Evidence shows that leaders permanently monitor instruction and provide educators with feedback and guidance. They plan and organize professional development to address specific instructional matters in addition to negotiating with local and regional administrators regarding the resources required to support professional development (Leithwood, 2010).

### 1.6 Relationships with the community

School development needs to be accompanied and supported by community, from the local to the regional and national levels. As past research recommends, efforts should focus on systemic capacity building, broadly extending to every school and every classroom (Harris, 2011). Well-trained professionals from district and national levels can facilitate and assess schools in their improvement processes, as their knowledge about the context of schools is useful in developing context-specific approaches, particularly for those facing challenging circumstances. Besides, networks of schools and teachers support each other's learning by visiting other schools and classes and by interchanging experiences and practices. Research has demonstrated that when interdependence and successful collaboration is established at the district level, professional learning networks improve, and collective capacity building is achieved (Boateng, 2014; Chapman et al., 2010; Fullan, 2010; Knipprath and Arimoto, 2007; Leithwood, 2010).

## 2 Method

### 2.1 Purpose

This study assesses the academic centers’ predictors and their relative effect on the perception the teaching staff has on the educational quality provided by the school.

### 2.2 Sample

This study is part of the research project EDU 2009-14773-C02 (“The impact of the implementation of quality systems in educational centers”), funded by the Spanish Ministry of Economy and Competitiveness.

The study sample consists of 14 non-university educational centers from the Basque Country, which have been part of the national study, providing 316 participants, of which 14% were members of the management team or quality managers, and 86% were teachers.

For the selection criteria of this study, centers should have implemented either one of the following quality management systems (QMS): the European Foundation for Quality Management (EFQM) or the Integrated Quality Project (PCI, for its Spanish acronym). In particular:

• 6 centers had implemented the EFQM model.
• 8 centers had implemented the PCI model.

The average length of service of the 14 centers was 50 years, the oldest being 130 years old and the newest 3 years old.

The QMS model has been implemented for 7.4 years average. Of the 14 participating schools, 8 had been awarded or prized for quality.

On average, the sample participants were 44.47 years old, with a mean service of 16.21 years at the center.

The sample consisted of 14 centers and 316 participants, of which 43 belonged to the center management team and 273 were part of the teaching staff (faculty). The average years of service at the centers is 16.21 (6.26), with teaching experience ranging from 0 to 41 years old.

The size of the participating educational centers varied between 49 and 1,668 students, with a mean of 930 (standard deviation; SD = 870). Due to the implementation process of the educational quality management models, school centers have received a mean of 1.6 awards (SD = 2), although, at the time of the study, 29.4% of the centers had not received any award, and 31.2% of them had been awarded with over three prizes.

### 2.3 Tool

The instrument used for data collection was known as Education Management Quality Assessment Instrument (IVCGE, for its Spanish acronym), which was designed jointly by the Innova research team at the University of Deusto and the Complutense University of Madrid, showing excellent results for its construct validity dimensions and weighing accuracy (reliability) through two confirmatory factor analyses (Villa et al., 2015). The instrument's reliability was assessed through Cronbach's alpha, thus using the SPSS19 and achieving excellent reliability (α = .955). Finally, its construct validity was assessed by means of the structural equations modeling, achieving highly satisfactory values (CMIN/DF = 4.831, IFI = .917, RMSEA = .057, PRATIO = .926).

After conducting an extensive bibliographical review of the QMS and schools, the design of the instrument (IVCGE) was configured by two major axes regarding the quality of a center, which have been shown to be interrelated.

The first axis—the quality systems policies—has three dimensions:

- Teaching planning: This competence can be defined as the efficient determination of the aims, priorities, methods, and controls to conduct activities in accordance with the deadlines and the means set.
- Communication: It constitutes the essential support at the educational center to conduct the organization's core functions.
- Acknowledgment: It is extremely important for the teaching staff and the school.

The second axis—the great processes implemented at the centers—measured through three dimensions:

- The organizational environment: It is assessed by the quality of the relationships between the members of the school and the feelings of acceptance and rejection of others.
- The teaching-learning process: It is a determining factor and considered the most pedagogical from the other dimensions, with its relevance being evidenced in many research works.
- The relationship with the environment: This assesses the role of the school and the relations established not only with the parents but also with the social environment surrounding it.

Due to conducting interviews of the participating staff members of the 14 educational centers, 316 questionnaires were collected. Then, the statistical analysis of the results was conducted.

A priori, it was based on two different models. One is the EFQM Model, which is from the business field and implemented in larger centers (1,000–1,500 students); this has more years of experience, multiple awards for quality, and a strong institutional support. The other model, PCI (Martínez and Galíndez, 2003, Sarasola et al., 2003, Villa et al., 2004), comes from the educational area, is applied at small centers (100–200 students), and has less years of experience, less awards, and no institutional support.

### 2.4 Procedure

To conduct the empirical study, a letter was sent to 24 educational centers (invited sample), to each Director in particular, requesting their participation. Fourteen responded affirmatively (participating sample), dismissing the involvement of the rest for various reasons such as lack of time, failure to meet the minimum requirements, rejection of the proposal, or not being willing to deliver the self-assessment. The letter ensured confidentiality when processing data, as well as the voluntary participation in the study, without discriminating the sample. Simultaneously, three requirements were demanded: the provision of a copy of the self-assessment report conducted by the center, a copy of the evaluation including the scores obtained by the external committee in the system used at the center (EFQM or PCI), and the provision of the existent team plans for improvement at the center.

Once the centers consented to participate in the study, a visit to the center was agreed upon, where teachers were given a 128-item questionnaire featuring a rating scale of 1 (lowest value) to 5 (highest value). Some were key-type questions so if the answer was “yes,” they continued answering with a rating scale from 1 to 5, and if the answer was “no,” the respondent moved on to the next question.

Finally, the centers were visited thrice in the same year, the data provided by the teaching staff were collected and used, an interview was conducted with the headmaster and the management team, and a final report was sent to each center. A letter of appreciation was sent to thank them for participating in the study.

### 2.5 Data analysis

The mean (M) and SD were used to describe the scalar variables and, in the case of nominal variables, the frequency (n) and percentage (%) were implemented.

To assess the objective of the study, a multiple linear regression model was used for each of the six dimensions of the IVCGE and for the total scale. The size of the effect is expressed in standardized beta coefficients (β) and the variance is explained by the model through the determination coefficient (R^2^).

### 2.6 Findings

**Figure 1:**
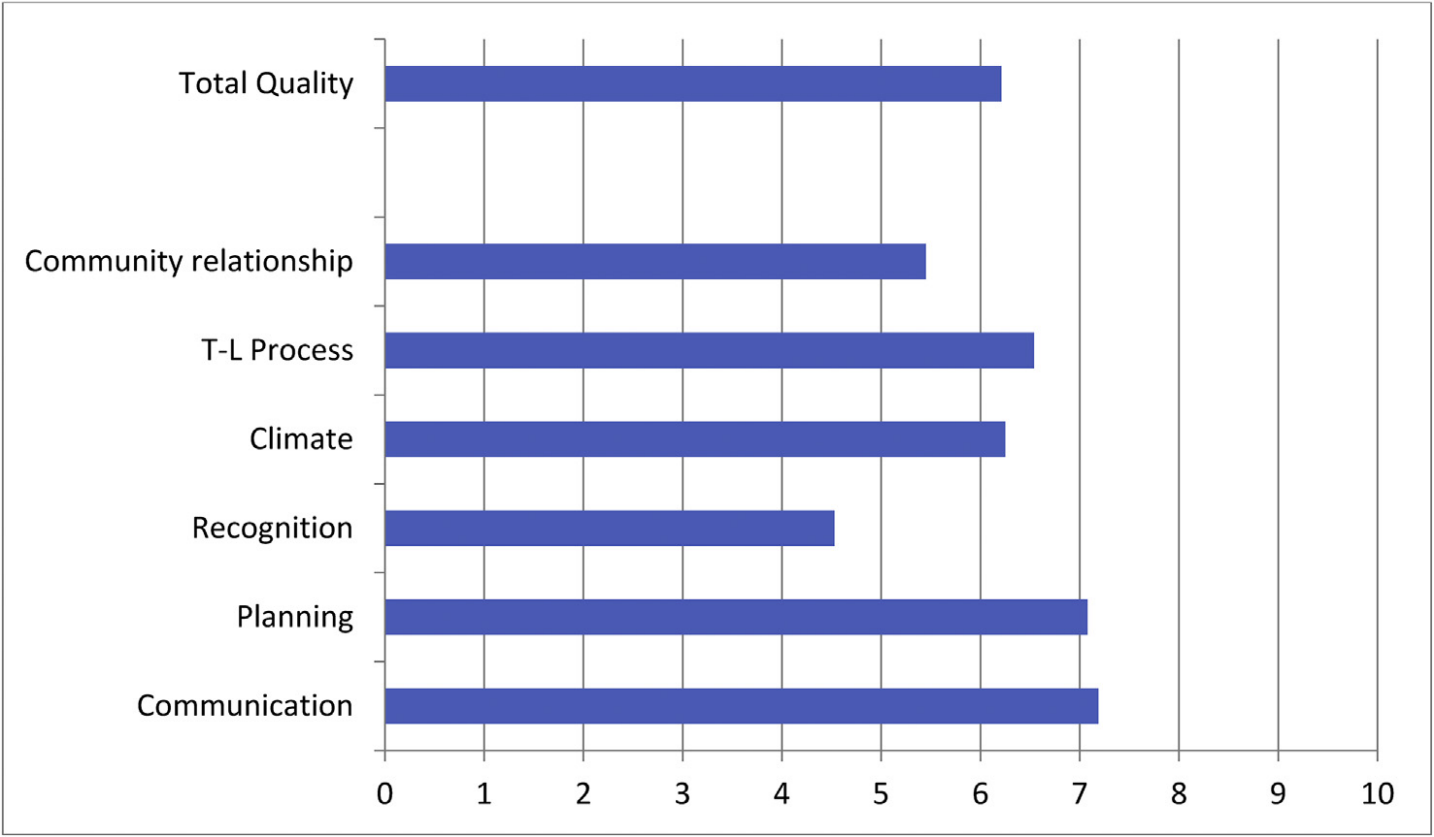
Mean scores observed for each dimension assessed by the IVCGE scale and the total quality score.

Figure 1 shows the mean scores observed for each dimension assessed by the IVCGE scale and the total quality score. In all cases, the possible values ranged from 0 to 10, without showing noteworthy ceiling or floor effects (extreme scores in percentage exceeding 5%).

The scores given by the participants regarding the quality of their educational projects varied from a minimum of 1.74 points to a maximum value of 9.63, with an average rating of 6.21 (SD = 1.74). The communication and planning dimensions are rated the best (M > 7), whereas the relationship with the community (M = 5.45) and teacher recognition and support (M = 4.53) obtained the lowest scores (see Table 1).

**Table 1:** Quality management system analysis model.

Dimension 1. Educational policies	Educational planning
	Communication
	Support and rewards for teachers
Dimension 2. Management processes	Organizational climate
	Teaching and learning process
	Relationships with the community

Note: authors' elaboration.

Table 2 shows the results of the seven regression models implemented for each of the six IVCGE quality dimensions, as well as the scale's total indicator. All the models were statistically significant, although with variance explained of small size (R^2^ = .13 for the Communication dimension), to moderate-low (R^2^ = .24, .25, and .28 for the climate, recognition, and total scale dimensions, respectively).

**Table 2:** Identification of the predictors of the IVCGE quality dimensions. Multiple Regression Analysis.

	Communication		Planning		Recognition		Climate		T-L Process		Community Relation		Total Quality	
	β	p	β	P	β	p	β	p	β	p	β	P	β	p
Position (Manager)	.07	.209	.24	.001	.08	.128	.16	.004	.08	.163	.01	.928	.12	.028
SGC Plan (PCI)	.51	.001	.74	.001	.41	.001	.54	.001	.56	.001	.21	.027	.59	.001
Nº of Prizes	.54	.001	.60	.001	.48	.001	.62	.001	.54	.001	.39	.001	.66	.001
Center Size	.25	.007	.42	.001	.42	.001	.34	.001	.38	.001	.24	.015	.41	.001
Seniority	-.02	.828	.07	.429	-.10	.243	-.05	.530	-.02	.830	.02	.856	-.03	.756
Age	-.02	.795	-.06	.489	.08	.300	.02	.779	-.01	.917	-.01	.960	.02	.838
F	7.55		12.71		16.26		15.57		10.65		9.63		18.29	
p	.001		.001		.001		.001		.001		.001		.001	
R2	.13		.21		.25		.24		.18		.16		.28	

Note. - β: Standardized regression coefficient; p: probability value; F: ANOVA test; R2: Determination coefficient/Explained variance.

Under no circumstance were the years of service of the teacher/employee or his/her age associated with the quality assessment of the educational program, and the position held by the respondent only shows a statistically significant effect for the planning (β = .24), climate (β = .16) dimensions, or for the total score (β = .12).

The variables number of awards received, implementation of a quality plan, and center size are shown to be statistically significant predictors in all the criteria variables, although the effect on them is not the same.

In the case of the total quality indicator, the main predictors are number of awards received (β = .66) and implementation of a quality plan (β = .59), followed by center size (β = .41) and, with a lower effect, the job held by the respondent (β = .12). The same can be observed in the climate, teaching-learning process, and communication dimensions. However, in the case of the teacher recognition and support, the number of awards (β = .48), the center size (β = .42), and the implementation of the quality program (β = .41) show a comparable effect.

Finally, in the case of the planning dimension, the main predictor in the implementation of the plan has a high effect (β = .74), the number of awards received has a moderate-high effect (β = .60), followed by the center size (β = .42) and the position held by the respondent (β = .24), with a moderate effect.

## 3 Discussion

In the last few decades, educational reforms have evolved in terms of handling tensions between central control from administration and the autonomy of schools (Boateng, 2014; Chapman et al., 2010; Knipprath and Arimoto, 2007). Fullan (2009) considered that at the international level, educational reforms have undergone different stages where accountability is a flagship for monitoring and fostering school improvement. However, this accountability culture and the systems that it has established have jeopardized the quality and equity of schools, particularly when league tables, standards, and competition have grown in importance (Ainscow, 2010; Harris, 2011; Mosoge and Pilane, 2014).

This study, along with other research on educational reforms, underlines that accountability measures need to go hand in hand with other strategies to achieve progress in students’ learning and performance, as this is the central purpose of educational systems (Creemers and Kyriakides, 2009; Hopkins et al., 2014). The new developments perceived in system reforms focus on four major factors that can improve the response of educational institutions: teacher quality and reward, quality and capacity building, transparency and accountability for results, and leadership (Fullan, 2009; Harris, 2011). Thus, educational reforms become the impetus that schools need to develop.

The application of a high-quality system in any organization and in education is a priority. Educational centers require the recognition of their pedagogical mission to distinguish themselves from other schools and to provide and promote adequate teaching and learning processes in an increasingly demanding and complex context.

From this study, some characteristics of this quality process can be underlined. As perceived in the findings, two factors regarding the dimensions of educational policies are significantly well valued: educational planning and communication. Schools can make real efforts to promote these key factors within their organization and with the local community. Principals and senior management teams can focus on planning and communicating with their educational staff, students, and families, allowing them to well understand their work, strategies, and objectives.

As several authors have underlined, the schools that are embarked in planning processes for improvement aim at fostering students’ learning with effective teaching and leadership. To this end, the authorities in charge must first clarify their goals and “develop a widely shared set of beliefs and vision about student achievement” (Leithwood, 2010, p. 6). This clear mission focused on students is supported by institutional financial allocations, personnel policies and procedures, and organizational structures. Moreover, school policies, cultures, and practices can tackle and overcome inequalities (Hopkins et al., 2014).

Meanwhile, evidence highlights that leaders in charge of decision-making and managing schools can develop transformational roles guiding the ongoing changes. Leadership functions are delegated among different members of the educational community, which, in turn, empowers professionals and institutions. Besides, leaders are devoted to improving teaching and learning. Instructional leaders have high expectations and support both teachers and students to achieve their respective goals (Camburn et al., 2003; Hopkins et al., 2014; Hoque et al., 2011).

Another aspect of this study that has been highlighted as relevant to improve quality in educational centers and is closely related to leadership is the work being developed to foster interpersonal communications, particularly among school staff, and among students and their families. Within educational communities, collaborative and collegial working procedures promote the fact that educators generate, exchange, and challenge their teaching practices. Thus, when teachers feel empowered and involved in decision-making regarding their students’ learning processes, they improve their pedagogy and develop professionally (Knipprath and Arimoto, 2007). With the aim of supporting these networks for self-reflection and continuous learning, educational systems are provided with structures and professionals that articulate and facilitate these relationships within and among schools in their process of becoming autonomous learning communities (Chapman et al., 2010; Hopkins et al., 2014).

Furthermore, as per the findings of this study, support and reward structures for teachers comprise the factor that is the least valued by this study's participants. Recognition is an external process that depends on several factors; it can come from models that are supported and even financed by public administrations, or even only from an agency that accredits the model. This has a lower social impact, regardless of the model's own added value. This means when a center receives accreditation of a model, the perception of the recognition varies because of the support received from the public administration in charge. As has been underlined, reward structures and support for teachers have a significantly lower value than the other factors. This is a matter of concern in terms of the crucial role of principals and senior management in the recognition and support that must be provided to teachers to make them feel motivated and involved in the educational community.

As several authors have stated, the actual impact of existing awards differs significantly from teachers’ aspirations, depending on various aspects of the prize: pedagogical enhancement, focus and organizational model, application requirements, evaluation criteria, and the distribution of outcomes. Accordingly, the most important objectives for establishing teaching and learning enhancement prizes are motivating academic staff for high-quality teaching, encouraging innovation in teaching and learning activities, and improving institutional recognition and awareness about teaching and learning enhancement (Efimenko et al., 2018).

Moreover, the improvement of quality in teaching and learning appears achieved only if schools establish appropriate professional development and reward structures motivating staff to become immersed in the changing processes. Therefore, school managers and leaders should agree on an improvement strategy that consider show the quality of teaching and learning should be enhanced. In addition, professional learning opportunities become accessible to provide educators with the technical skills required. Efforts are rewarded and praised, fostering professional involvement and sustaining capacity building in schools (Fullan, 2010; Hopkins et al., 2014; Leithwood, 2010).

Considering the second dimension related to management processes, organizational climate, teaching and learning processes, and relationships with the community were analyzed. The research findings indicate a strong significant difference in school climate and the perception of teaching quality at different educational levels. A decline in the perception of teaching quality and school climate is apparent. Regarding higher education, teaching is becoming more focused on achievements and less focused on pedagogical processes. Moreover, the study's findings support the claim that school climate and teaching quality contribute to learning achievements. It can be assumed that achievement-focused teaching, without considering the quality of teaching and a good school climate, will not ensure students' success at school (Magen-Nagar and Azuly, 2016).

Thus, the results of this study confirm a significant difference when a good school climate is perceived and when the perception of the quality of teaching is high. Furthermore, an important factor is the leadership of an educational center. Although the behavior of teachers, students, and parents contributes to school climate, the attitude and actions of principals and senior management are crucial to maintaining a favorable climate in the school. Their behavior can either prevent or promote a positive atmosphere. On the one hand, teachers rely on school principals for motivation, management, and development. On the other hand, students depend on school leadership to guarantee quality education. Moreover, parents perceive principals as those who maintain high academic standards, as well as the identity of the school, so that they are assured that their children are receiving the best education available (Rapti, 2015).

A crucial factor in the perception and evaluation of the educational quality of a center is the teaching and learning processes, which were highly valued by teaching staff in this study. Considering pre-service teacher training, in a recent study, Netshifhfhe et al. (2017) conclude that quality assurance should be an integral part of teaching and learning in universities. All teaching and learning activities from curriculum planning to assessment should be based on ways to enhance quality. The issue of quality is important as universities seek to remain relevant by producing graduates that fit well in society and serve to drive its socio-economic and political functions. Academics in universities should understand and embrace the concept of quality assurance to be accountable in their teaching because accountability is relevant to all stakeholders, particularly to students. Therefore, quality assurance should be a professional exercise rather than an externally driven management initiative.

In our findings, relationships with community were given less value by the teachers who were involved in the study. Therefore, it is necessary to train teachers to know how to foster their relationships with community owing to the important effect that it has on the better development of schools. School-to-school networks contribute to the construction of communities of practice and collaborative structures, locally and regionally, fostering improvement at three levels: classroom, school, and system. Thus, lateral capacity building is developed by fostering collective responsibility on instruction, sharing a moral purpose, and learning with and from one another. Hence, schools acquire a coherent, consistent holistic view and strategy for the improvement of all parts of their establishment and of the educational system as a whole (Ainscow, 2010; Chapman et al., 2010; Fullan, 2010; Stoll, 2009).

In conclusion, we would like to highlight that recent research has emphasized the use of evidence for school planning, organizational learning, and accountability (Leithwood, 2010). Knipprath and Arimoto (2007) highlight data as a valuable tool to measure quality in education and to assure a certain level of achievement throughout the entire process: prescribing standards (input), monitoring implementation (process), and checking the generation of desired results (output). Evidence suggests that establishing external and internal evaluation mechanisms has a strong effect in school improvement, particularly where the teaching quality in classrooms is poor (Creemers and Kyriakides, 2009). Implementation of self-evaluation processes help educational institutions to identify priorities for development, analyze potential for improvement, and foster accountability (Vast'atková, 2010). Besides, some authors have stressed the potential benefits of data, particularly when staff become involved in enquiry and reflection activities that enrich school planning and teaching practices (Ainscow et al., 2000). Therefore, further research on quality systems implemented in schools should shed light on the aspects to be evaluated to promote educational transformation that will, in turn, improve the teaching practices of learning professionals and organizations.

## Declarations

### Author contribution statement

F. Díez: Conceived and designed the experiments; Contributed reagents, materials, analysis tools or data; Wrote the paper.

A. Villa: Performed the experiments; Contributed reagents, materials, analysis tools or data; Wrote the paper.

I. Iraurgi: Analyzed and interpreted the data; Contributed reagents, materials, analysis tools or data.

A.L. López: Contributed reagents, materials, analysis tools or data; Wrote the paper.

### Funding statement

This research did not receive any specific grant from funding agencies in the public, commercial, or not-for-profit sectors.

### Competing interest statement

The authors declare no conflict of interest.

### Additional information

No additional information is available for this paper.
